# Study of the Stability, Uptake and Transformations of Zero Valent Iron Nanoparticles in a Model Plant by Means of an Optimised Single Particle ICP-MS/MS Method

**DOI:** 10.3390/nano13111736

**Published:** 2023-05-25

**Authors:** Justyna Wojcieszek, Sandrine Chay, Javier Jiménez-Lamana, Catherine Curie, Stephane Mari

**Affiliations:** 1 Chair of Analytical Chemistry, Faculty of Chemistry, Warsaw University of Technology, Noakowskiego 3, 00-664 Warsaw, Poland; 2 Centre for Advanced Materials and Technologies CEZAMAT, Warsaw University of Technology, Poleczki 19, 02-822 Warsaw, Poland; 3 IPSiM, Université de Montpellier, CNRS, INRAE, Institut Agro, Place Viala, CEDEX 1, 34060 Montpellier, France; sandrine.chay@inrae.fr (S.C.); catherine.curie@cnrs.fr (C.C.); stephane.mari@inrae.fr (S.M.); 4 Universite de Pau et des Pays de l’Adour, E2SUPPA, CNRS UMR 5254, IPREM, 64053 Pau, France; j.jimenez-lamana@univ-pau.fr

**Keywords:** zero-valent iron nanoparticles, model plant, single particle inductively coupled plasma mass spectrometry, histochemical procedures, accumulation, transformations

## Abstract

In the context of the widespread distribution of zero valent iron nanoparticles (nZVI) in the environment and its possible exposure to many aquatic and terrestrial organisms, this study investigates the effects, uptake, bioaccumulation, localisation and possible transformations of nZVI in two different forms (aqueous dispersion—Nanofer 25S and air-stable powder—Nanofer STAR) in a model plant—*Arabidopsis thaliana*. Seedlings exposed to Nanofer STAR displayed symptoms of toxicity, including chlorosis and reduced growth. At the tissue and cellular level, the exposure to Nanofer STAR induced a strong accumulation of Fe in the root intercellular spaces and in Fe-rich granules in pollen grains. Nanofer STAR did not undergo any transformations during 7 days of incubation, while in Nanofer 25S, three different behaviours were observed: (i) stability, (ii) partial dissolution and (iii) the agglomeration process. The size distributions obtained by SP-ICP-MS/MS demonstrated that regardless of the type of nZVI used, iron was taken up and accumulated in the plant, mainly in the form of intact nanoparticles. The agglomerates created in the growth medium in the case of Nanofer 25S were not taken up by the plant. Taken together, the results indicate that *Arabidopsis* plants do take up, transport and accumulate nZVI in all parts of the plants, including the seeds, which will provide a better understanding of the behaviour and transformations of nZVI once released into the environment, a critical issue from the point of view of food safety.

## 1. Introduction

With the development of nanotechnology, iron-based nanoparticles (NPs) are increasingly applied in numerous fields due to their better physicochemical properties compared with bulk particles, such as a high specific surface area and great sorption sites [1,2]. Fe-based NPs are especially used in biomedical and environmental industries, for groundwater and soil remediation, and in agriculture to improve plant growth and Fe nutrient accumulation in plants [3,4,5,6,7]. However, while nanotechnology is advancing rapidly in many areas of life sciences, the proper applications of NPs in plant and agricultural research are still lagging [8]. As a result of their extensive use, Fe-based NPs are released into the environment and can have an impact on living organisms. Although iron is considered to be a non-toxic element, a growing number of studies indicate negative effects of iron-based NPs on living organisms [1,9]. Moreover, after being released into the environment, iron-based NPs usually undergo various transformations, such as aggregation, dissolution, interactions with macromolecules and redox reactions, which influence their fate, transport and potential toxicity. In a real, complex environment, iron-based NPs can undergo different transformations at the same time, which calls for more insightful studies [1,10,11].

Among iron-based nanoparticles, zero-valent iron nanoparticles (nZVI) are recognized to be excellent adsorbents and effective photocatalysts. For that reason, nZVI have gained special interest because of their efficiency in the treatment of several pollutants and their toxic metabolites, especially for chlorinated solvents and metal/metalloids, leading to their use for in situ remediation of groundwater and soil. The advantages of nZVI are their low cost, great effectiveness, large surface area, non-toxicity and enormous flexibility for in situ applications [3,12,13,14,15,16]. However, treating contaminated water and soil with nZVI is considered to be the largest single stream of engineered NPs into the environment, suggesting that field scale operations need at least 3 g·L^−1^ of nZVI for efficient degradation of contaminants [1,17]. As a result, the accumulation of nZVI in soils and its exposure to plants and animals is likely to occur. Additionally, nZVI is highly reactive and its toxicity and bioavailability may change upon different transformation processes. Consequently, bioavailable transformed species may be taken up by flora and fauna, raising serious concerns about their bioaccumulation and biomagnification in food chains and the eventual impact on humans [18,19,20]. Therefore, there is a pressing need to improve our knowledge and understanding of the existing gaps in the research of nanoparticles, particularly the bioaccumulation of nanomaterials and the potential risks associated with them [8].

There are few possibilities of nZVI uptake and transport in plants. From a growth medium or soil, nZVI can penetrate the walls of plant root cells, enter the epidermal cells, and pass through the cortex. Another route of passage is through the epidermis and cortex via apoplastic pathways. Finally, nZVI can be taken up and transported to stems and leaves via the xylem and phloem [21,22]. In the case of foliar exposition, nZVI can first be absorbed by stomata or trichomes on the leaves, and then moved to the roots via phloem vessels [23,24]. Once taken up and accumulated, nZVI can interact with plants at the cellular and subcellular levels, facilitating changes to the plants’ morphological and physiological states, which can be either suppressive or stimulating. Both the toxic and beneficial effects of nZVI on plant tissues have been already reported. The responses of plants to nZVI exposure have been presented to be dependent on nZVI concentration, but the effect of nZVI on plant growth was also found to depend on the plant species and the medium used for plant cultivation [25,26,27,28]. Low concentrations of nZVI can promote plant growth, as it was reported, for example, in the case of *Typha latifolia* [28] or peanut seedlings [29], suggesting that an appropriately chosen concentration of nZVI can be used for the remediation of contaminated water and soils without harmful effects on the environment. In the case of the peanut plant, low concentrations of nZVI not only provoked the growth of the plant, but were also associated with seed germination by penetrating the peanut shells in order to increase water uptake [29]. Two grass species exhibited no symptoms of toxicity under nZVI application, and even biomass production and root growth increased, especially in the case of *A. capillaris* [30]. Positive effects of plant growth were observed even for high concentrations of nZVI [31,32]. On the other hand, a negative impact of nZVI on the germination and growth of different plants has been reported, suggesting that, in addition to oxidative stress, the inhibiting effect of nZVI is assigned to the blocking of water and nutrient uptake by plant roots, which is caused by direct deposition and accumulation of nZVI on the root surface [25,27,28,33]. Moreover, nZVI translocation can affect antioxidant enzymes’ activities and alter the active iron content [34]. However, the environmental impact of nZVI on plants is still far from being well understood. The potential hazards associated with the presence of nZVI in the environment must be fully characterised in order to mitigate the possible negative effects on the environment and the health of organisms [35,36].

In the context of the widespread production and distribution of nZVI in different fields and the possible risk of their exposure to living organisms, this study aimed at multiple objectives: (i) the investigation of the localisation of iron accumulated in plant tissues of *Arabidopsis thaliana* exposed to nZVI through histochemical procedures (Perls and Perls/DAB protocol); (ii) the optimisation of reaction cell conditions for ICP-MS analysis in MS/MS mode for the determination and characterisation of nZVI in plant tissues; (iii) the study of the stability of nZVI in the nutrient medium by tandem ICP-MS in single particle mode (SP-ICP-MS/MS); and (iv) the identification of the physico-chemical form and the size characterisation of nZVI in two different forms (aqueous dispersion and air-stable powder) accumulated in *Arabidopsis thaliana* by SP-ICP-MS/MS. To the best of our knowledge, this is the first time that a combination of single particle ICP-MS/MS with histochemistry has been used for the study of the uptake, localisation and transformations of nanoparticles in a model plant.

## 2. Materials and Methods

### 2.1. Samples and Reagents

Analytical or biological reagent-grade chemicals and LC-MS grade solvents were purchased from Sigma-Aldrich (St. Louis, MO, USA), unless stated otherwise. Ultra-pure water (18 MΩ cm) obtained with a Milli-Q system (Millipore, Guyancourt, France) was used throughout this work. The nitric acid of purity for trace metal analysis was purchased from Fluka (Buchs, Switzerland). Standard ionic solutions of 1000 mg L^−1^ iron and gold were purchased from Agilent Technologies (Tokyo, Japan). Macerozyme R-10 enzyme (pectinase from *Rhizopus* sp., Sigma Aldrich) was used to digest the plant tissues for nZVI extraction. Macerozyme R-10 is a multicomponent enzyme mixture containing cellulase (0.1 unit per mg), hemicellulase (0.25 unit per mg) and pectinase (0.5 unit per mg).

Commercially available nZVI were used in the study in order to create conditions relevant to the surrounding environment of a real remediation site. Nanofer 25S slurry, an aqueous dispersion of 20 wt% iron nanoparticles with a special organic (PAA) surface modification, and Nanofer STAR, an air-stable nZVI powder, were supplied by NANO IRON, s.r.o. (Rajhrad, Czech Republic). nZVI in the form of aqueous dispersion and in the form of powder will be referred to hereinafter as Nanofer 25S and Nanofer STAR, respectively. A stock suspension of Nanofer STAR was prepared by dispersion of the powder in ultrapure water. Diluted suspensions of Nanofer STAR and Nanofer 25S were prepared daily in ultrapure water by accurately weighing aliquots of the stock suspension after one minute of sonication using a Bandelin Sonorex DT 52 H ultrasonic bath (BANDELIN Electronic, Berlin, Germany). A gold nanoparticle (AuNPs) suspension standard with a nominal diameter of 50 nm was purchased from BBI Solutions (Cardiff, UK). All the suspensions of nanoparticles were stored in the dark at 4 °C and sonicated directly before SP-ICP-MS/MS analysis.

### 2.2. Plant Cultivation

For the biological experiments, *Arabidopsis thaliana* plants (ecotype Col-0) were cultivated either in soil or in half Murashige and Skoog medium, 1.2% (*w*/*v*) agar in petri dishes without sucrose, and with either Fe-EDTA (50 µM, standard conditions) or with Nanofer STAR suspension as the Fe source. Two different ranges of Nanofer STAR concentrations were used: (i) lower concentrations, from 1 to 50 mg L^−1^, which are equivalent to standard conditions and (ii) higher concentrations, from 0.2% to 1% (*w*/*v*) nZVI, corresponding to concentrations from 2 to 10 g L^−1^. The Nanofer 25S was not used in these experiments to avoid the potential phytotoxicity of the stabilizing compounds (PAA) used to keep the NPs in suspension. For that reason, the Nanofer 25S was exclusively used for short-term exposure of young seedlings (see below). The medium was buffered with 2.5 mM MES at pH 5.7. For all the in vitro experiments, the plants were cultivated in growth chambers with a light intensity of 80 µmol m^−2^ s^−1^, with an 8 h night/16 h day photoperiod and 65% relative humidity at 22 °C. For the soil experiments, the plants were grown in a greenhouse (22 °C) in soil supplemented with Nanofer STAR at concentrations from 0.2% to 1% (*w*/*v*) nZVI. The plants were cultivated for two months, which is the duration required for the plants to complete their cycle and produce seeds.

Taking into account the high sensitivity of the ICP-MS technique, an additional set of plants was cultivated for experiments carried out by ICP-MS/MS and SP-ICP-MS/MS (see Section 2.5), with Nanofer 25S and Nanofer STAR at lower concentrations (5 mg L^−1^ and 8 mg L^−1^, respectively). For these experiments, the seedlings were cultivated in the in vitro culture media as described above, except that the agar was omitted to maintain the liquid state of the media. The seedlings were cultivated under gentle shaking for 7 days, harvested, washed with distilled water and freeze-dried.

### 2.3. Determination of Total Iron Content

Samples of each variant of *Arabidopsis thaliana* plant (0.05 g) cultivated in nutrient medium were digested by the TOPEX+ microwave digestion system (Preekem Scientific, Perlan Technologies, Warsaw, Poland) with a mixture of 5 mL HNO_3_ (c) and 2 mL of H_2_O_2_ (c). After digestion, the mixtures were cooled and diluted with ultrapure water to a final volume of 20 mL. Further dilutions were prepared with 2% HNO_3_ (*v*/*v*) directly before ICP-MS/MS analysis. The determination of the total concentration of iron in the plant samples was carried out using an Agilent 8900 ICP-MS/MS (Agilent Technologies, Tokyo, Japan) instrument fitted with Pt cones and a 2.5 mm i.d. injector torch. The position of the torch and nebuliser gas flow was adjusted each day of work. The working conditions of ICP-MS/MS were optimised daily using a 1 µg L^−1^ solution of ^7^Li^+^, ^89^Y^+^ and ^205^Tl^+^ in 2% (*v*/*v*) HNO_3_. Three isotopes of iron (^54^Fe, ^56^Fe, ^57^Fe) were monitored during the analysis. The analytical blanks were analysed in parallel. Quantification was performed by external calibration: a 7-point calibration curve was prepared for the iron concentration in the investigated range from 0.0 to 50.0 µg L^−1^. The content of iron in each sample was calculated as the mean of the results obtained for the monitored isotopes from three replicates of each sample. 

In the case of the plants cultivated in soil, Fe was quantified by atomic emission spectroscopy with a microwave plasma atomic emission spectrometer (MP-AES, Agilent Technologies). Briefly, the samples were dried at 80 °C for 2 days. For mineralisation, the tissues were digested completely in 70% (*v*/*v*) HNO_3_ and 30% (*v*/*v*) H_2_O_2_ for 16 h at 85 °C in a mineralisation ramp (Hotblock). Elemental analysis was performed by MP-AES using the calibration standard solutions provided by the manufacturer.

### 2.4. Iron Staining by Perls and Perls/DAB Histochemical Procedures

Iron staining by the Perls method and Perls with DAB intensification were performed according to Roschzttardtz et al. [37], both on the whole seedlings and on the histological sections from the roots and flowers.

### 2.5. Enzymatic Digestion Method

After cultivation was performed on the *Arabidopsis thaliana* seeds, and prior to the SP-ICP-MS/MS analysis, the plant tissues were digested enzymatically, as reported in previous works [38,39] with some modifications. The previously used metal ultrasonic probe provoked a significant iron contamination in the samples, as reported in a previous study with titanium dioxide nanoparticles [40]. Therefore, grounded samples of the plants (0.015 g) were homogenized with 7 mL of 2 mM citrate buffer (pH 4.5; adjusted with citric acid) by using a KIMBLE Dounce tissue grinder set of 7 mL (Sigma-Aldrich) made of glass. Afterwards, 1.5 mL of Macerozyme R-10 solution (0.02 g of enzyme powder dissolved in 1.5 mL of ultrapure water) was added to the samples, and they were then incubated at 37 °C for 24 h with continuous shaking at 400 rpm by means of a MultiTherm^TM^ shaker with heating and cooling (Benchmark, Lodi, NJ, USA). After digestion, the samples were allowed to settle for approximately 60 min and the obtained supernatants were then diluted with ultrapure water and analysed by SP-ICP-MS/MS.

### 2.6. Single Particle ICP-MS/MS Method

An Agilent 8900 ICP-MS/MS equipped with an SPS-4 Series Autosampler and Single Nanoparticle Application Module was used for the characterisation of nZVI. The default instrumental and data acquisition parameters are listed in Table S1. During the analysis, ^56^Fe with a natural abundance of 91.7% was monitored. To overcome possible spectral interferences at *m*/*z* 56 (^40^Ar^16^O^+^, ^40^Ca^16^O^+^), H_2_ was introduced as a reaction gas, which interacts with the interfering ions. Analyses were performed in “on-mass” mode, e.g., the first and the third quadrupole were set to *m*/*z* 56. SP-ICP-MS/MS analyses were performed in TRA mode using a dwell time of 100 µs, with a total time of analysis of 120 s. Working with microsecond dwell times allows for the recording of particle events as resolved transient signals, thereby improving the resolution and working range. A gold nanoparticle (AuNPs) standard with a nominal diameter of 50 nm was used for the determination of the transport efficiency, which was calculated by the particle size method described by Pace et al. [41]. The sample flow rate was calculated daily by measuring the mass of water taken up by the peristaltic pump for 2 min (this operation was repeated 3 times). Under the experimental conditions used in this work, the nebulisation efficiency at a sample flow rate of 0.35 mL min^−1^ was 7.5%. The suspensions of nZVI were appropriately diluted with ultrapure water in order to detect a sufficient number of nanoparticles during analysis and to avoid multiple particle events. Before the sample analysis, an EDTA solution was analysed to eliminate the memory effect. Similar to our previous work, the samples were not filtered prior to analysis, as only some of the nanoparticles were detected in the recovered filtrates [40]. After each sample analysis, the software automatically processed the raw data and generated the particle size, particle concentration, size distribution and information about the concentration of the dissolved metal. The discrimination between the dissolved signal and the nanoparticle signal was determined on the basis of the threshold (I_thresh_); it was determined as I_thresh_ = I_b_ + 5σ_b_, where I_b_ is the intensity of the baseline and σ_b_ is the standard deviation of the baseline of each sample. A criterion of 5σ_b_ was recently considered to be a good compromise between keeping the rate of false positives as low as possible, while at the same time, not omitting a significant number of the true particle event [42].

### 2.7. Statistical Analysis

The data were presented as the mean values of at least three replicates with standard deviation (SD). Significant differences between the treatments were performed using the one-way analysis of variance (ANOVA). The differences were considered as statistically significant when the *p*-value was <0.05. The size distributions were prepared in Origin 8.5 software (Northampton, MA, USA) and adjusted to lognormal distributions in order to obtain the median diameter.

## 3. Results and Discussion

### 3.1. Responses of Arabidopsis Thaliana Seedlings to nZVI Exposure in Nutrient Medium

**Figure 1:**
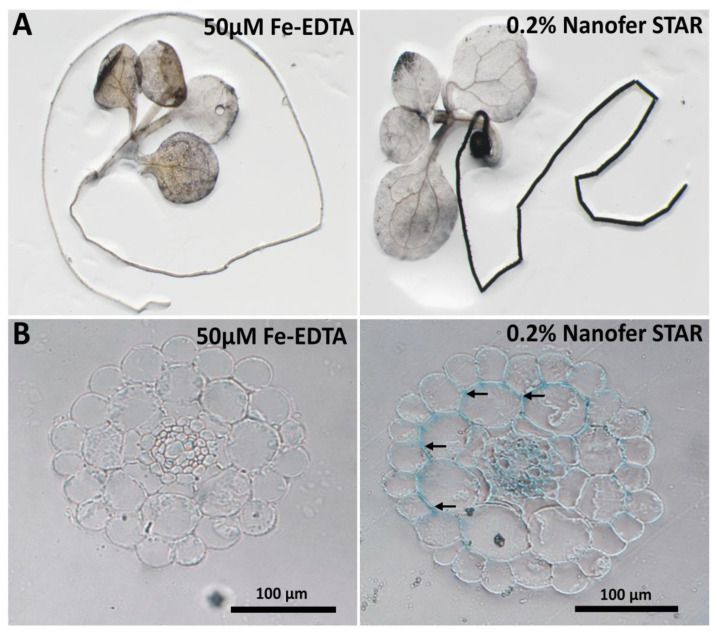
Histochemical staining of Fe with (**A**) Perls/DAB procedure (Fe is stained in brown-black) on 14-day-old seedlings grown in 50 µM Fe-EDTA (standard condition) or in 0.2% (*w*/*v*) nZVI, and with (**B**) Perls procedure (Fe is stained in blue) in resin sections from roots of 14-day-old seedlings grown in 50 µM Fe-EDTA or in 0.2% nZVI (*w*/*v*).

In order to investigate the effect of nZVI on plants, *Arabidopsis thaliana* seedlings were cultivated for 14 days in a solid nutrient medium supplemented with either a standard source of Fe (i.e., Fe-EDTA) or increasing concentrations of Nanofer STAR. In the first step, Nanofer STAR concentrations comparable to the standard medium were used for plant cultivation, ranging from 1 to 50 mg L^−1^ Fe (Figure S1). The exposure to such low concentrations of nZVI did not induce any visible beneficial or toxic effect, and, more importantly, the corresponding concentrations of Fe were not sufficient to be further visualized by histochemical staining [37]. Therefore, in the next step, higher nZVI concentrations were used in the nutrient medium, ranging from 0.2% to 1% (*w*/*v*), which corresponds to concentrations from 2 to 10 g L^−1^. The exposure to higher concentrations of Nanofer STAR resulted in a reduction of the root length and increased chlorosis (yellowing of leaves due to decreased accumulation of chlorophyll), in a dose-response manner (Figure S2). To gain more insight on the impact of nZVI on plants, the whole seedlings were stained for Fe using the Perls/DAB histochemical procedure that stains Fe atoms in tissues brown-black. Compared to the control plants, where Fe was mostly detectable in the leaf veins (Figure 1A, left panel), the exposure to 0.2% nZVI led to a massive accumulation of Fe in the roots (Figure 1A, right panel). For a more detailed analysis of Fe localisation within the roots, the roots were fixed and embedded in resin, and the corresponding thin sections were stained for Fe with K-ferrocyanide (Perls reagent), which reveals Fe as a blue colour (the Perls reagent alone is less sensitive but more quantitative than the Perls/DAB). As it is presented in Figure 1B, in comparison to the control, the roots exposed to 0.2% nZVI showed intensive iron accumulation in the intercellular space between the epidermal and cortex cells (marked with arrows) and also in the central cylinder, strongly suggesting that, to some extent, nZVI had been accumulated within the root tissues and that some Fe had been transferred to the conductive elements for the translocation to the above-ground parts of the plant.

Taken together, the obtained results indicate that Nanofer STAR appears to be toxic to plants in the higher concentrations and that the nanoparticles can be accumulated at the root surface or even between the first layers of cells. The presence of intense Fe staining in the central cylinder indicated that some iron, whether in the form of nZVI or in other forms, had passed through all the cell layers and was transported towards the upper parts of the plant seedling.

### 3.2. Long-Term Exposure to Nanofer STAR Applied on Soil

**Figure 2:**
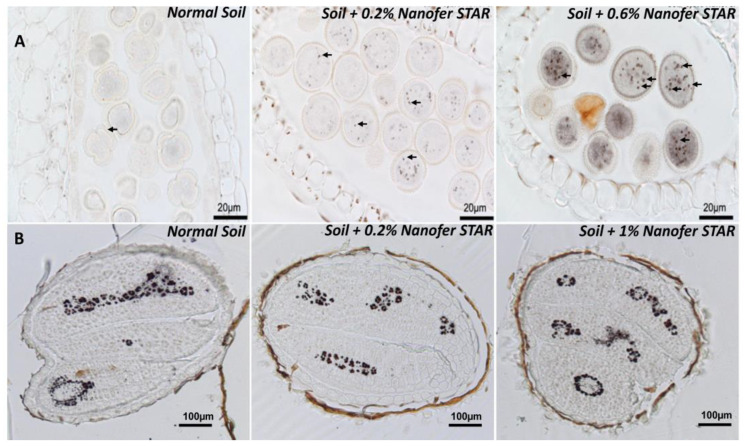
Fe Perls/DAB staining on thin sections of resin-embedded (**A**) pollen grains in anthers (male organ of the flower), (**B**) dry seeds, both from plants grown on soil and exposed to the indicated concentrations of nZVI. Arrows in (**A**) indicate Fe-rich structures within pollen grains. Black structures in (**B**) correspond to Fe stored in vacuoles of specific cells that surround the provascular strands in the embryo.

On the basis of these first findings, the impact of nZVI on longer exposures was monitored in order to assess the potential effects on a whole plant cycle (i.e., until the plants set seeds). For this purpose, *Arabidopsis thaliana* plants were cultivated in a greenhouse in soil supplemented with Nanofer STAR at the same concentrations as in the previous experiment. In comparison to the in vitro experiments, the same concentrations of Nanofer STAR applied directly to the soil (environmentally realistic exposure scenario) did not lead to visible symptoms of toxicity in the plant (or beneficial growth effect). In the case of a different commercially available nZVI, Yoon et al. [43] showed that the presence of nZVI in soil, even at a high concentration, benefits shoot growth and enhances photosynthesis, suggesting that nZVI has the potential to be used as a nano-fertilizer. Afterwards, the total content of iron in the leaves, floral stems and seeds harvested from these plants was determined by microwave plasma atomic emission spectroscopy (MP-AES). The obtained results showed that exposure to nZVI provoked a two- to three-fold increase in the iron concentration in the leaves, and a two-fold increase in the stems, indicating that, to some extent, some of the Fe carried by nanoparticles was taken up by the plants and translocated to the upper organs (Figure S3). The next step focused on the flowers, more precisely, on the anthers, which are the organs responsible for the production of pollen that carry the male genetic material. Whole flowers from the control and nZVI-treated plants were fixed, embedded in resin, sectioned and stained with Perls/DAB. Strikingly, the exposure to nZVI did provoke some visible Fe accumulation in the anther cells and the pollen grains (Figure 2A, marked with arrows), compared to the control flowers. Moreover, this Fe accumulation was even more pronounced for the plants treated with 0.6% nZVI. In this case, the pollen grains showed an intense and uniform grey staining in the cytosol together with very intense Fe deposits, which were not observed in the pollen from the control plants (Figure 2A, right panel). These results indicate that the exposure to nZVI did provoke a massive Fe overload in all the above-ground organs, affecting even the reproductive organs. The chemical identity of these Fe deposits within the pollen grains remains to be determined, along with whether or not they correspond to intact nZVI that have been translocated from the roots to the flowers.

The exposure to nZVI had a much more moderate effect on the total Fe concentration in seeds, since it increased the iron content and provoked a 23 to 32% increase in the Fe concentration (Figure S4), suggesting that the plants could regulate and protect seeds from Fe overaccumulation in the leaves. Afterwards, the Fe was stained in sections from dry seeds harvested after the nZVI treatments in order to determine whether the exposure to nanoparticles could have induced some particular accumulation of Fe in the seed tissues. When compared to the control seeds that stored Fe in the vacuoles of provascular cells, the seeds from the nZVI-treated plants showed exactly the same Fe distribution pattern, indicating that, although seeds had accumulated more Fe, the final storage of Fe had not been affected by this treatment (Figure 2B).

### 3.3. Optimisation of H_2_ Gas Flow for ICP-MS Analysis in MS/MS Mode

In order to identify the form of iron taken up and accumulated in plant tissues, further experiments were carried out by SP-ICP-MS/MS. The feasibility of SP-ICP-MS is compromised by the achievable size detection limits, which is critically affected by both the instrument response toward the isotope monitored and on the background signal which is dependent on the interferences of the instrument and the content of the dissolved species of the element measured. The higher the background signal, the lower the capability of detecting small nanoparticles, hence the higher the size limit of detection [44]. Iron has four naturally occurring stable isotopes with abundances from 0.3 to 91.7% [45], which are interfered mainly by Ar- and Ca-containing ions [46]. In order to obtain the highest sensitivity, the monitoring of the most abundant isotope of iron (^56^Fe—91.7%) during SP-ICP-MS analysis appears as a natural choice. However, this requires the removal of polyatomic interferences coming from plasma (^40^Ar^16^O^+^) and a sample matrix (^40^Ca^16^O^+^) [47]. The use of ICP-MS in the MS/MS mode provides the possibility to overcome the interferences of iron by introduction of a cell gas (H_2_), which interacts with the interfering ions. Hydrogen has previously been applied effectively as a cell gas for selective removal of Ar and other plasma matrix ions [48,49,50].

**Figure 3:**
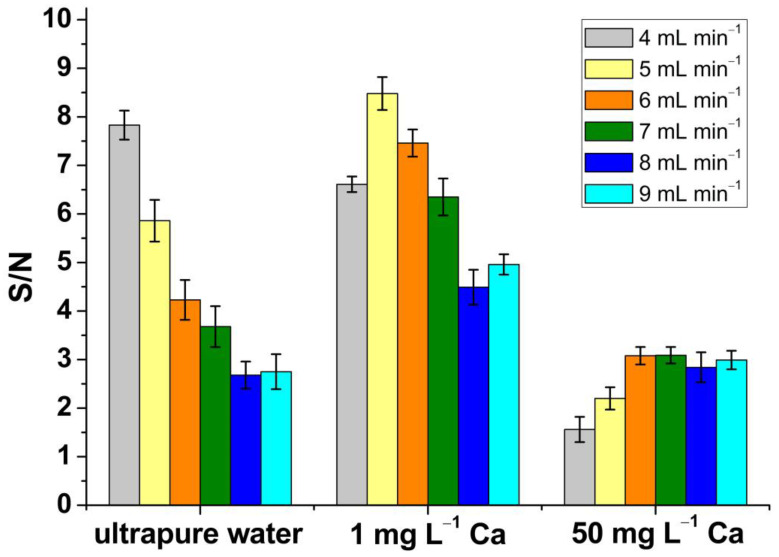
Signal-to-noise ratio obtained for three different matrices: ultrapure water, 1 mg L^−1^ Ca and 50 mg L^−1^ Ca at different hydrogen gas flows (4–9 mL min^−1^). Error bars represent the standard deviation of the measurement determined for 5 replicates.

The method was first optimised, and attention was paid to the H_2_ gas flow. In order to obtain the highest signal-to-noise ratio, a 1 ppb Fe solution was analysed by SP-ICP-MS/MS using different flows of H_2_ and obtaining the instrument response towards the analyte. The analysis was performed “on-mass” with both quadrupoles set up to *m*/*z* 56 [50]. Subsequently, three matrices were also analysed: ultrapure water, 1 mg L^−1^ Ca and 50 mg L^−1^ Ca, and the corresponding signals obtained were treated mathematically in order to obtain the associated noise, calculated as δ. Afterwards, the signal-to-noise ratio was obtained. Figure 3 shows the signal-to-noise ratio obtained using H_2_ flows from 4 to 9 mL min^−1^.

The best values were obtained with H_2_ flows of 4 mL min^−1^ for ultrapure water, 5 mL min^−1^ for 1 mg L^−1^ Ca and 6 mL min^−1^ for the matrix containing 50 mg L^−1^ Ca. However, since a significant amount of Ca is expected in plant tissues (up to 0.1–5% of dry weight [51]), a H_2_ flow of 6 mL min^−1^ was retained for the rest of the study. When H_2_ was used at a flow of 6 mL min^−1^, the most effective removal of the polyatomic interferences was observed in the calcium-rich matrices. The H_2_ gas flow chosen for nZVI characterisation was in good agreement with another study based on the investigation of nZVI behaviour in wastewater by SP-ICP-MS/MS [48].

### 3.4. Total Content of Iron in Arabidopsis Thaliana Plant

In order to study the iron uptake by plant tissues, the determination of the total iron content in *Arabidopsis thaliana* was performed after plant cultivation in nutrient medium (for ICP-MS/MS analysis, see Section 2.2) in different conditions: control plants, plants grown in the presence of nZVI as dispersion (Nanofer 25S, at a concentration of 5 mg L^−1^), as powder (Nanofer STAR, at a concentration of 8 mg L^−1^) and in the presence of Nanofer STAR together with an iron complex with EDTA. The last variant was performed in order to verify if the presence of iron ions in addition to nZVI, applied at the same concentration as for the cultivation with Nanofer STAR alone, could affect the total content of iron accumulated in the plant. The control and NP-treated samples of the plant were analysed by standalone ICP-MS/MS after the samples’ mineralization. The content of iron in each sample was calculated as the mean of results obtained for the monitored isotopes from three replicates of each sample. The obtained results are presented in Table 1.

The samples treated with nZVI showed a significantly higher iron content than those obtained for the control plants (significant difference in one-way ANOVA, level of confidence > 95%), showing that iron is taken up and accumulated in plant tissues. The highest content of iron was observed in plants cultivated with Nanofer 25S, despite the lower concentration of nZVI used for plant cultivation compared to Nanofer STAR, suggesting that iron is more available for the plant when it is directly present in a suspension. Therefore, the efficiency of the accumulation of iron in plant tissues is dependent on its form supplied to the plant. Additionally, a higher iron content was observed in the case of plants treated only with Nanofer STAR as compared to the plants cultivated with the Nanofer STAR together with iron complexed with EDTA. This could indicate some type of competition between the ionic and NPs form of metal, leading to a lower accumulation of iron. It could be also explained by an agglomeration process of nZVI in the presence of iron ions, as has already been documented in other studies [52]. As it has already been demonstrated, NP agglomerates created in a growth medium during cultivation are not taken up by plants [53]. Taking into account that a significant amount of nZVI is intentionally released into the environment for in situ soil and groundwater remediation, effective accumulation of iron in plant tissues after their exposure to nZVI should be considered from the point of view of food safety. This is especially important due to the high reactivity of nZVI, which can lead to possible changes in its toxicity and bioavailability in different environmental processes.

### 3.5. Characterisation of nZVI Suspensions and Size Detection Limit

In SP-ICP-MS/MS, the size detection limit (*LOD_size_*) is critically dependent on the standard deviation of the intensity of the signal corresponding to the background and/or to the dissolved fraction of the analysed element (*σ_B_*). According to Laborda et al. [54], the size detection limit can be calculated from Equation (1):

(1) $${LOD}_{size}={\left(\frac{6\times 3\sigma_{B}}{\pi \rho X_{NP}K_{ICPMS}K_{M}}\right)}^{\frac{1}{3}}$$

where *ρ* is the density of the nanoparticles; *X_NP_* is the mass fraction of the element in the nanoparticles; *K_ICPMS_* is the detection efficiency (ratio of the number of ions detected to the number of atoms introduced into the ICP), which is dependent on the particular instrument; and *K_M_* (=*AN_Av_*/*M_M_*) includes the contribution from the element measured (*A*, the atomic abundance of the isotope considered; *N_Av_*, Avogadro’s number; *M_M_*, the atomic mass). It is difficult to assess the value of *K_ICPMS_*; however, the term *K_ICPMS_K_M_* can be calculated from Equation (2):

(2) $$K_{ICPMS}K_{M}=\frac{R}{K_{intr}C^{M}}$$

where *R* represents the ions counted per time unit; *K_intr_* (=*η_neb_Q_sam_*) is related to the contribution from the sample introduction system through the nebulization efficiency (*η_neb_*) and the sample uptake rate (*Q_sam_*), whose values can be easily calculated as described in the Materials and Methods section; and *C^M^* is the mass concentration of a solution of an analyte nebulized into an ICP-MS.

By monitoring the most abundant isotope, ^56^Fe, working with H_2_ as the reaction gas and a dwell time of 100 μs, a size detection limit of 38 nm could be achieved. However, it should be mentioned that the *LOD_size_* was calculated for ultrapure water as a matrix. Taking into account a real, complex matrix of plant samples, the contribution of iron ions is usually significant, which leads to a higher background and, thus, a higher size detection limit of the nanoparticles.

Before the analysis of the plant samples cultivated with nZVI, Nanofer 25S and Nanofer STAR, at a nominal size of 50 nm, were characterised by SP-ICP-MS/MS with the optimised cell gas conditions. The corresponding time scans and size distributions obtained are shown in Figure S5. For both cases, a significant number of peaks characteristic of the presence of nZVI was observed on the time scans. The obtained size distributions showed a polydisperse distribution of nZVI, with a main peak at a size close to the nominal diameter of the nZVI. The median diameters were determined at 56 and 59 nm for Nanofer 25S and Nanofer STAR, respectively. These results are in good agreement with the results obtained by TEM analysis, which have been presented by other authors. For example, Oprčkal et al. [55] showed that the diameter of Nanofer STAR and Nanofer 25 was in the range of 30–80 nm and 20–80 nm, respectively. Moreover, sharp narrow peaks observed for nZVI on the XRD patterns represented an α-Fe^0^ body-centred cubic cell (bcc) phase. TEM images presented by Zabetakis et al. [56] showed that, in the case of Nanofer STAR, the particles vary in size, but have an average diameter of 60 nm, and for Nanofer 25S, the particles average 55 nm in diameter and are surrounded by an amorphous material, most likely the organic surface coating. Vidmar et al. [48] demonstrated via TEM that the primary diameters of fresh nZVI particles (Fe core) ranged from 20 to 80 nm, with 4- to 6-nm-thick shells. An XRD analysis further demonstrated that the nZVI had well-defined crystalline core structures, in which a zero valent iron (α-Fe0) body-centred cubic cell (bcc) phase prevailed. Mirlohi S. [57] used the DLS technique for characterisation of nZVI, and the particle size distribution of Nanofer 25S ranged from 18 to 110 nm, with an average size of 52 nm. On the other hand, Nanofer STAR behaved more like micro- rather than nanoparticles, with the average size estimated at 635 nm. However, it should be mentioned that similar results were obtained by the SP-ICP-MS/MS technique when too high a concentration of Nanofer STAR suspension was analysed. In appropriately diluted suspensions, the determined diameters were in good agreement with the manufacturer’s data and other studies discussed here. In both cases, signal from the background, most likely derived from the dissolved metal fraction, was observed; however, the intensity of this signal did not hinder the efficient size characterisation of nZVI. 

### 3.6. Influence of Filtration on Size and Number of nZVI Detected by SP-ICP-MS/MS

Before analysis by SP-ICP-MS/MS, it is recommended to prevent the introduction of larger particles into the system, which may result in the blocking of the nebuliser or in the contamination of the inner parts of the instrument. For this reason, filtration is often used with the expectation that 0.45 µm membranes will not remove significant quantities of 1–100 nm NPs. Despite the use of filters with pore sizes substantially exceeding the nanoscale, particle retention can occur, leading to an inaccurate quantification of NP concentrations in natural systems [58,59]. Therefore, the impact of filtration on the number of nanoparticles detected during SP-ICP-MS/MS analysis was investigated before the analysis of the plant samples. Suspensions of Nanofer 25S and Nanofer STAR at 2.5 ± 0.2 × 10^7^ and 2.1 ± 0.1 × 10^7^ NPs L^−1^, respectively, were filtered through 0.45 µm filters with PTFE membrane and analysed by SP-ICP-MS/MS. As it is shown in Table 2, only about 50% of nZVI was detected in the recovered filtrates, which can be explained by the agglomeration of NPs and/or their adsorption on the membrane surface and pore walls. In addition, the size distributions of the filtered suspensions of nZVI showed lower median diameters for both dispersion and powder of nZVI compared to the stock suspensions, suggesting that larger NPs are more likely to be retained in filters.

These results are in good agreement with other studies in which authors have reported a retention of 70% of nZVI on 0.45 µm pore size filters [48]. For this reason, the plant samples were not filtered before SP-ICP-MS/MS analysis. After enzymatic digestion, the samples were allowed to settle by gravity for 1 h, and only the upper part of the suspension was utilized for further analysis, avoiding the pipetting of the solid plant matrix.

### 3.7. Investigation of nZVI Stability in Growth Medium

Metal-containing NPs are reactive species that can interact with various chemical compounds. In this context, the stability of NPs in the growth medium used for plant cultivation needs to be investigated before the cultivation and analysis of the plants. This step is essential in model studies of NPs–plants interactions in order to elucidate if the possible transformations of NPs take place before or after their uptake by plants [53]. In order to determine whether nZVI undergo transformations in the growth medium used, the influence of exposure time was verified. For this purpose, the solution of medium used for plant cultivation was spiked with Nanofer 25S and Nanofer STAR and analysed immediately upon the addition of nZVI and over time. Each analysed suspension was diluted accordingly in order to maintain the same theoretical number of NPs and, hence, detect the same number of events. It is worth mentioning that in addition to Nanofer 25S and Nanofer STAR, both with a modified surface in order to enhance their stability, Nanofer 25, an uncoated nZVI, was characterised by SP-ICP-MS/MS. The time scan registered immediately after the preparation of the Nanofer 25 suspension showed a fast dissolution of nZVI with only a few detected nanoparticles, which led to the conclusion that modification of the nZVI surface is necessary to prevent immediate transformations of NPs after their contact with chemical components.

**Figure 4:**
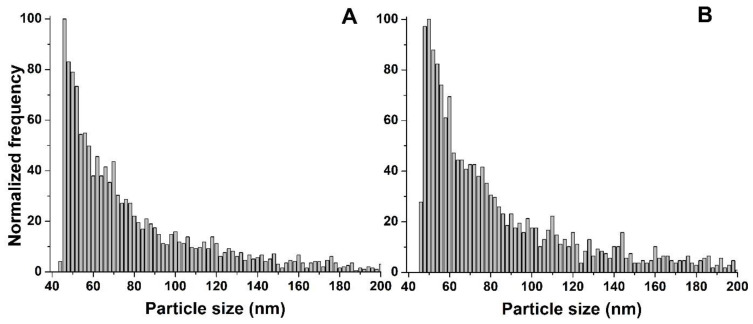
Size distributions of Nanofer STAR in the growth medium (**A**) freshly prepared and (**B**) after 7 days of incubation.

In the case of Nanofer STAR, the size distributions obtained from the freshly prepared suspension and after 7 days of incubation are presented in Figure 4.

No significant differences were observed in the size distributions obtained from the freshly prepared mixture and after 7 days of incubation, with median nZVI sizes of 59 and 61 nm, respectively. Moreover, no significant background signal was observed on the SP-ICP-MS/MS time scans, regardless of the incubation time studied. Taking into account the high reactivity of nZVI, their interactions with environmental components could easily lead to different transformations, such as dissolution or agglomeration. However, the surface of iron nanoparticles in Nanofer STAR is specially stabilized in order to prevent changes in its structure, and the obtained results show that nZVI in the form of powder are stable in the growth medium and do not undergo any transformations until the end of the plant cultivation.

**Figure 5:**
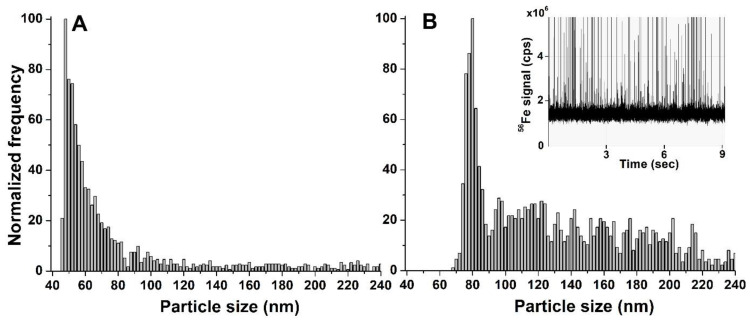
Size distributions of Nanofer 25S in the growth medium (**A**) freshly prepared and (**B**) after 7 days of incubation (part of the SP-ICP-MS/MS time scan is in the insert).

However, a different situation was observed for the nZVI dispersion. In general, there are three possible scenarios regarding the behaviour of NPs during the plant cultivation: (i) NPs remain unchanged in the medium, (ii) NPs are partially or totally dissolved or (iii) NPs agglomerate over time. All three possible scenarios were observed during the investigation of the stability of Nanofer 25S in the growth medium. The size distributions obtained after the SP-ICP-MS/MS analysis of a freshly prepared solution and after 7 days of incubation are presented in Figure 5. As can be observed from the figure, NPs that were intact were still present in the growth medium after 7 days of incubation. Simultaneously, a significant background signal coming from the metal in its ionic form was also detected in the time scan (Figure 5B insert), suggesting that, in addition to the presence of intact NPs, some of them underwent partial dissolution. Due to the high background signal, the size detection limit of nZVI after 7 days of incubation is significantly higher in comparison with the freshly prepared suspension. Finally, additional signals at higher sizes were observed in the size distribution after 7 days of incubation, indicating that some of the nZVI in the form of dispersion also agglomerated. This second distribution was not observed for the freshly prepared suspension, suggesting that the NPs underwent agglomeration over time. This resulted in a higher median nanoparticle diameter (81 nm) after 7 days of incubation compared with the freshly prepared suspension (55 nm). Although the surface of Nanofer 25S is also modified to prevent their transformations, the obtained results showed significantly impaired stability of the nZVI dispersion in comparison with air-stable powder.

Taking into account all of the possible scenarios, i.e., stability, dissolution and agglomeration, which were observed when studying the behaviour of NPs in the growth medium, the next step was focused on the determination of the final form of iron taken up and accumulated in *Arabidopsis thaliana* tissues.

### 3.8. Single Particle ICP-MS/MS Analysis of Arabidopsis Thaliana Treated with nZVI

In order to identify the form of iron in *Arabidopsis thaliana,* the plant samples treated with Nanofer 25S and Nanofer STAR were subjected to an enzymatic procedure, followed by SP-ICP-MS/MS analysis under optimised conditions of H_2_ gas flow. After enzymatic digestion, the supernatants were appropriately diluted with ultrapure water to detect a sufficient number of particles during each measurement (at least 300) and to avoid multiple particle events, and then analysed by SP-ICP-MS/MS. Much greater dilutions had to be made for the plants cultivated with Nanofer 25S, which confirmed the results of the determination of the total iron content, suggesting that *Arabidopsis thaliana* accumulated iron much more effectively when supplied in the form of nZVI dispersion. On the time scans obtained for the plants treated with nZVI, a significant number of signals characteristic of the presence of NPs can be observed, indicating the ability of the plant to uptake and accumulate iron as intact NPs (Figure S6). The presence of nZVI in the plant tissues has been already documented for poplar root cells [28]. For plants cultivated with Nanofer 25S, a non-significant signal originating from the ionic form of metal was also observed. The total nanoparticle number concentration determined in the plants cultivated with nZVI dispersion and powder was 2.6 ± 0.4 × 10^13^ and 6.0 ± 0.8 × 10^11^ NPs g^−1^, respectively.

**Figure 6:**
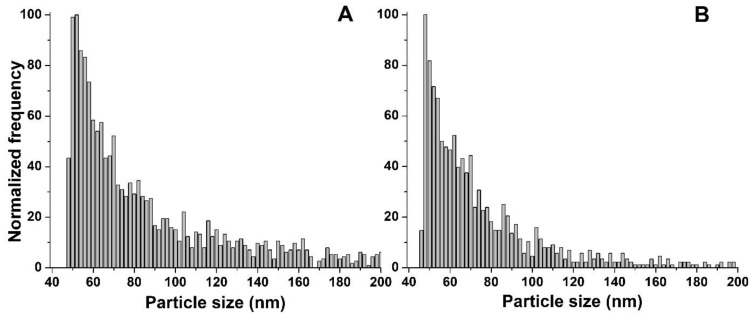
Size distributions obtained after SP-ICP-MS/MS analysis of plants treated with (**A**) Nanofer 25S and (**B**) Nanofer STAR.

The size distributions obtained after an analysis of the plants cultivated with both forms of nZVI, presented in Figure 6, showed the polydisperse distribution of NPs, with median diameters of 59 nm for both Nanofer 25S and Nanofer STAR (Table 3). One-way ANOVA revealed no significant differences, with a level of confidence > 95%, between the determined diameters of nZVI accumulated in *Arabidopsis thaliana* and the Nanofer 25S and Nanofer STAR stock suspensions.

For Nanofer STAR, no significant differences were observed in the size distribution obtained after an analysis of the plant, compared to the one obtained for the nZVI standard stock suspension and for the suspension after 7 days in the growth medium. This led to the conclusion that unchanged nZVI were taken up and accumulated in the plant, i.e., no transformations, such as dissolution or agglomeration, took place. The obtained results showed that, from the investigation of the nZVI form in the cultivation medium to the characterisation of NPs extracted from the plant tissues, nZVI in the form of powder demonstrated themselves to be stable and resistant to changes in their structures.

In the case of nZVI in the form of dispersion, the stability study in the growth medium showed three different behaviours of nZVI, which led to the consideration of the final form of iron accumulated in the plant. The majority of pulses detected during the SP-ICP-MS/MS analysis of the plant were characteristic for the presence of intact NPs, indicating the stability of nZVI after their uptake and accumulation inside the plant. Moreover, after an analysis of the plant tissues, the second peak at larger sizes derived from the agglomerates of NPs was not observed in the size distributions, suggesting that they were not taken up by the plant, which is in good agreement with our previous experiences [40,60]. Additionally, although a partial dissolution of Nanofer 25S was observed after 7 days of incubation in the growth medium, a much lower amount of ionic iron was obtained in the sample of the plant treated with the nZVI suspension, indicating that the presence of plant tissue may affect the form of accumulated metal. Transformation of iron ions can take place inside the plant in the form of NPs or some biominerals that behave in the same way as NPs during SP-ICP-MS/MS analysis. This type of conversion from ionic iron to biominerals such as iron phosphates has already been described in other studies [61,62,63]. However, the majority of the signals were observed at sizes around 50 nm, which is a nominal diameter of the analysed nZVI, suggesting that iron is taken up and accumulated in *Arabidopsis thaliana* tissues mainly in the form of intact nanoparticles. It is also worth mentioning that higher concentrations of nZVI can lead to their aggregation and adhesion to the root surface, which can block water and nutrient uptake by plant tissues, leading to plant death. In the case of experiments carried out by SP-ICP-MS/MS, the lower concentration of nZVI used for plant cultivation did not cause toxic effects and allowed the determination of nZVI taken up and accumulated inside the plant tissues. The results obtained for plants cultivated with Nanofer 25S showed that although three different forms of iron were observed in the nutrient medium, mainly intact nZVI are eventually taken up and accumulated in plant tissues. It is worth highlighting that nZVI are the sole engineered NPs that have been injected into the ground in large quantities and dispersed with groundwater flow, possibly exposing themselves to many aquatic and terrestrial organisms. Taking into account the high-dose inhibition effects of nZVI on the growth and development of plants, the obtained results are important from the point of view of nanotechnology safety and could be helpful for obtaining a better understanding of the nZVI fate after their release into the environment, as well as their impact on living organisms.

## 4. Conclusions

This study investigated the effects, stability, uptake, bioaccumulation, physicochemical characterisation, transformations and localisation of widely used zero valent iron nanoparticles in *Arabidopsis thaliana* tissues by means of Fe imaging (histochemistry) and single particle ICP-MS/MS. 

When tested in vitro, a higher concentration of the Nanofer STAR induced symptoms of toxicity that could be, at least in part, due to the massive accumulation of Fe in the roots. However, when applied directly on the soil, similar to conditions in the field, the Nanofer STAR did not provoke any visible symptom of toxicity, despite the fact that the exposure was much longer in this case (up to 2 months) and that the plants did accumulate higher concentrations of Fe. The observation in the pollen grains of unusual Fe-rich deposits that had never been reported before, even in plants treated with an excess of ionic Fe, strongly suggests that the nanoparticles had been taken up by the plants and transported and accumulated in all the organs.

After confirming the latter by ICP-MS/MS, the use of SP-ICP-MS/MS allowed us to obtain information about the physicochemical form of the iron accumulated in the plants. The study of the stability of nZVI in the growth medium showed that Nanofer STAR was stable until the end of the plant cultivation. In the case of Nanofer 25S, processes of partial dissolution and agglomeration of NPs were additionally observed after 7 days of cultivation. An SP-ICP-MS/MS analysis of the plant tissues after enzymatic treatment showed the size distributions of nZVI to be in good agreement with data provided by the manufacturer, leading to the conclusion that mainly intact nZVI are taken up and accumulated in the plant. In the case of plants treated with Nanofer 25S, agglomerates of nZVI created in the growth medium were not taken up by the plant.

Taking into account the potential exposure of nZVI to many aquatic and terrestrial organisms, the present study will help provide a better understanding of the fate of nZVI after it is released into the environment, which is important from the point of view of food and environmental safety.

## Figures and Tables

**Table 1:** Total iron content in *Arabidopsis thaliana* from control and plants treated with Nanofer 25S, Nanofer STAR and Nanofer STAR together with Fe-EDTA. All results are expressed as mean ± SD determined for 3 samples, each measured 3 times.

	Iron Content/µg g^−1^
Controls (Fe-EDTA)	0.9 ± 0.1
Nanofer 25S	150.7 ± 10.3
Nanofer STAR	9.3 ± 0.4
Nanofer STAR + Fe-EDTA	6.7 ± 0.2

**Table 2:** Comparison of median diameters and number of detected NPs obtained for two nZVI suspensions before and after filtration through a 0.45 µm pore size filter. All results are expressed as mean ± SD determined for 5 replicates.

	Nanofer 25S		Nanofer STAR	
	Before Filtration	After Filtration	Before Filtration	After Filtration
Number of NPs detected	1220 ± 87	560 ± 73	1027 ± 27	530 ± 32
Diameter (nm)	59 ± 4	46 ± 2	61 ± 2	49 ± 1
NPs number concentration (NPs L^−1^)	2.5 ± 0.2 × 10^7^	1.1 ± 0.1 × 10^7^	2.1 ± 0.1 × 10^7^	1.1 ± 0.1 × 10^7^

**Table 3:** Median diameters, NPs number concentration and mass concentration of Nanofer 25S and Nanofer STAR obtained by SP-ICP-MS/MS for the analysis of *Arabidopsis thaliana* tissues after enzymatic treatment. All results are expressed as mean ± SD determined for 3 samples, each measured 3 times.

	Diameter (nm)	NPs Number Concentration (NPs g^−1^)	Mass Concentration (ng g^−1^)
Nanofer 25S	59 ± 1 *	2.6 ± 0.4 × 10^13^	1.1 ± 0.3 × 10^8^
Nanofer STAR	59 ± 2 *	6.0 ± 0.8 × 10^11^	3.0 ± 0.4 × 10^6^

* Diameters showing no significant differences with diameter obtained for stock suspension with a level of confidence > 95% (one-way ANOVA).

## Data Availability

The data presented in this study are available upon request from the corresponding author.

## Supplementary Materials

The following supporting information can be downloaded at: https://www.mdpi.com/article/10.3390/nano13111736/s1, Table S1: Default instrumental and data acquisition parameters for SP-ICP-MS/MS; Figure S1: Impact of Nanofer STAR exposure on seedlings grown for 7 days in low concentrations of nZVI; Figure S2: Impact of Nanofer STAR exposure on seedlings grown for 14 days in the indicated nZVI concentrations; Figure S3: Fe concentration in rosette leaves and floral stem from plants grown in soil supplemented with the indicated concentrations of Nanofer STAR (% nZVI, *w*/*v*); Figure S4: Fe concentration in dry seeds harvested from plants grown in soil supplemented with the indicated concentrations of Nanofer STAR (% nZVI, *w*/*v*). Error bars correspond to standard deviation of three replicates; stars indicate significant differences compared to control (Student’s test, *p* < 0.1); Figure S5: Time scans and size distributions of (A) Nanofer 25S and (B) Nanofer STAR obtained after SP-ICP-MS/MS analysis; Figure S6: Time scans obtained after SP-ICP-MS/MS analysis of plants treated with (A) Nanofer 25S and (B) Nanofer STAR.

## References

[1] Lei C., Sun Y., Tsang D.C.W., Lin D. (2018). Environmental transformations and ecological effects of iron-based nanoparticles. Environ. Pollut..

[2] Kumar S., Kumar M., Singh A. (2021). Synthesis and characterization of iron oxide nanoparticles (Fe_2_O_3_, Fe_3_O_4_): A brief review. Contemp. Phys..

[3] Yan W., Lien H.L., Koel B.E., Zhang W.X. (2013). Iron nanoparticles for environmental clean-up: Recent developments and future outlook. Environ. Sci. Process. Impacts.

[4] Liu G., Gao J., Ai H., Chen X. (2013). Applications and potential toxicity of magnetic iron oxide nanoparticles. Small.

[5] Landa P. (2021). Positive effects of metallic nanoparticles on plants: Overview of involved mechanisms. Plant Physiol. Biochem..

[6] Zhang Y., Goss G.G. (2022). Nanotechnology in agriculture: Comparison of the toxicity between conventional and nano-based agrochemicals on non-target aquatic species. J. Hazard. Mater..

[7] Walmsley G.G., McArdle A., Tevlin R., Momeni A., Atashroo D., Hu M.S., Feroze A.H., Wong V.W., Lorenz P.H., Longaker M.T. (2015). Nanotechnology in bone tissue engineering. Nanomed. Nanotechnol. Biol. Med..

[8] Murali M., Gowtham H.G., Singh S.B., Shilpa N., Aiyaz M., Alomary M.N., Alshamrani M., Salawi A., Almoshari Y., Ansari M.A. (2022). Fate, bioaccumulation and toxicity of engineered nanomaterials in plants: Current challenges and future prospects. Sci. Total Environ..

[9] Stefaniuk M., Oleszczuk P., Ok Y.S. (2016). Review on nano zerovalent iron (nZVI): From synthesis to environmental applications. Chem. Eng. J..

[10] Mitrano D.M., Motellier S., Clavaguera S., Nowack B. (2015). Review of nanomaterial aging and transformations through the life cycle of nano-enhanced products. Environ. Int..

[11] Dwivedi A.D., Ma L.Q. (2014). Biocatalytic synthesis pathways, transformation, and toxicity of nanoparticles in the environment. Crit. Rev. Environ. Sci. Technol..

[12] Crane R.A., Scott T.B. (2012). Nanoscale zero-valent iron: Future prospects for an emerging water treatment technology. J. Hazard. Mater..

[13] Rani M., Shanker U. (2018). Degradation of traditional and new emerging pesticides in water by nanomaterials: Recent trends and future recommendations. Int. J. Environ. Sci. Technol..

[14] Lefevre E., Bossa N., Wiesner M.R., Gunsch C.K. (2016). A review of the environmental implications of in situ remediation by nanoscale zero valent iron (nZVI): Behavior, transport and impacts on microbial communities. Sci. Total Environ..

[15] Fu F., Dionysiou D.D., Liu H. (2014). The use of zero-valent iron for groundwater remediation and wastewater treatment: A review. J. Hazard. Mater..

[16] Zhang W.X. (2003). Nanoscale iron particles for environmental remediation: An overview. J. Nanopart. Res..

[17] Chen J., Xiu Z., Lowry G.V., Alvarez P.J.J. (2011). Effect of natural organic matter on toxicity and reactivity of nano-scale zero-valent iron. Water Res..

[18] Grieger K.D., Fjordbøge A., Hartmann N.B., Eriksson E., Bjerg P.L., Baun A. (2010). Environmental benefits and risks of zero-valent iron nanoparticles (nZVI) for *in situ* remediation: Risk mitigation or trade-off?. J. Contam. Hydrol..

[19] Gardea-Torresdey J.L., Rico C.M., White J.C. (2014). Trophic transfer, transformation, and impact of engineered nanomaterials in terrestrial environments. Environ. Sci. Technol..

[20] Dwivedi A.D., Yoon H., Singh J.P., Chae K.H., Rho S., Hwang D.S., Chang Y.-S. (2018). Uptake, Distribution, and Transformation of Zerovalent Iron Nanoparticles in the Edible Plant *Cucumis sativus*. Environ. Sci. Technol..

[21] Zhang S., Yi K., Chen A., Shao J., Peng L., Luo S. (2022). Toxicity of zero-valent iron nanoparticles to soil organisms and the associated defense mechanisms: A review. Ecotoxicology.

[22] Stampoulis D., Sinha S.K., White J.C. (2009). Assay-dependent phytotoxicity of nanoparticles to plants. Environ. Sci. Technol..

[23] Ma C., White J.C., Dhankher O.P., Xing B. (2015). Metal-based nanotoxicity and detoxification pathways in higher plants. Environ. Sci. Technol..

[24] Da Silva L.C., Oliva M.A., Azevedo A.A., De Araujo J.M. (2006). Responses of restinga plant species to pollution from an iron pelletization factory. Environ. Sci. Pollut. Res. Int..

[25] Wang J., Fang Z., Cheng W., Yan X., Tsang P.E., Zhao D. (2016). Higher concentrations of nanoscale zero-valent iron (nZVI) in soil induced rice chlorosis due to inhibited active iron transportation. Environ. Pollut..

[26] Guha T., Ravikumar K.V.G., Mukherjee A., Mukherjee A., Kundu R. (2018). Nanopriming with zero valent iron (nZVI) enhances germination and growth in aromatic rice cultivar (*Oryza sativa* cv. Gobindabhog L.). Plant Physiol. Biochem..

[27] El-Temsah Y.S., Joner E.J. (2012). Impact of Fe and Ag nanoparticles on seed germination and differences in bioavailability during exposure in aqueous suspension and soil. Environ. Toxicol..

[28] Ma X., Gurung A., Deng Y. (2013). Phytotoxicity and uptake of nanoscale zero-valent iron (nZVI) by two plant species. Sci. Total Environ..

[29] Li X., Yang Y., Gao B., Zhang M. (2015). Stimulation of Peanut Seedling Development and Growth by Zero-Valent Iron Nanoparticles at Low Concentrations. PLoS ONE.

[30] Teodoro M., Clemente R., Ferrer-Bustins E., Martínez-Fernández D., Pilar Bernal M., Vítková M., Vítek P., Komárek M. (2020). Nanoscale Zero-Valent Iron Has Minimum Toxicological Risk on the Germination and Early Growth of Two Grass Species with Potential for Phytostabilization. Nanomaterials.

[31] Libralato G., Devoti A.C., Zanella M., Sabbioni E., Mičetić I., Manodori L., Pigozzo A., Manenti S., Groppi F., Ghirardini A.V. (2016). Phytotoxicity of ionic, micro- and nano-sized iron in three plant species. Ecotoxic. Environ. Saf..

[32] Kim J.H., Lee Y., Kim E.J., Gu S., Sohn E.J., Seo Y.S., An H.J., Chang Y.S. (2014). Exposure of Iron Nanoparticles to *Arabidopsis thaliana* Enhances Root Elongation by Triggering Cell Wall Loosening. Environ. Sci. Technol..

[33] El-Temsah Y.S., Sevcu A., Bobcikova K., Cernik M., Joner E.J. (2016). DDT degradation efficiency and ecotoxicological effects of two types of nano-sized zero-valent iron (nZVI) in water and soil. Chemosphere.

[34] Zhang R., Bai X., Shao J., Chen A., Wu H., Luo S. (2020). Effects of zero-valent iron nanoparticles and quinclorac coexposure on the growth and antioxidant system of rice (*Oryza sativa* L.). Ecotoxicol. Environ. Saf..

[35] Li M., Zhang P., Adeel M., Guo Z., Chetwynd A.J., Ma C., Bai T., Hao Y., Rui Y. (2021). Physiological impacts of zero valent iron, Fe_3_O_4_ and Fe_2_O_3_ nanoparticles in rice plants and their potential as Fe fertilizers. Environ. Pollut..

[36] Latif A., Sheng D., Sun K., Si Y., Azeem M., Abbas A., Bilal M. (2020). Remediation of heavy metals polluted environment using Fe-based nanoparticles: Mechanisms, influencing factors, and environmental implications. Environ. Pollut..

[37] Roschzttardtz H., Conéjéro G., Divol F., Alcon C., Verdeil J.-L., Curie C., Mari S. (2013). New insights into Fe localization in plant tissues. Front. Plant Sci..

[38] Jiménez-Lamana J., Wojcieszek J., Jakubiak M., Asztemborska M., Szpunar J. (2016). Single particle ICP-MS characterization of platinum nanoparticles uptake and bioaccumulation by *Lepidium sativum* and *Sinapis alba* plants. J. Anal. At. Spectrom..

[39] Kińska K., Jiménez-Lamana J., Kowalska J., Krasnodębska-Ostręga B., Szpunar J. (2018). Study of the uptake and bioaccumulation of palladium nanoparticles by *Sinapis alba* using single particle ICP-MS. Sci. Total Environ..

[40] Wojcieszek J., Jiménez-Lamana J., Ruzik L., Asztemborska M., Jarosz M., Szpunar J. (2020). Characterization of TiO_2_ NPs in radish (*Raphanus sativus* L.) by Single Particle ICP-QQQ-MS. Front. Environ. Sci..

[41] Pace H.E., Rogers N.J., Jarolimek C., Coleman V.A., Higgins C.P., Ranville J.F. (2011). Determining transport efficiency for the purpose of counting and sizing nanoparticles via single particle inductively coupled plasma mass spectrometry. Anal. Chem..

[42] Laborda F., Gimenez-Ingalaturre A.C., Bolea E., Castillo J.R. (2019). Single particle inductively coupled plasma mass spectrometry as screening tool for detection of particles. Spectrochim. Acta Part B At. Spectrosc..

[43] Yoon H., Kang Y.G., Chang Y.S., Kim J.H. (2019). Effects of Zerovalent Iron Nanoparticles on Photosynthesis and Biochemical Adaptation of Soil-Grown *Arabidopsis thaliana*. Nanomaterials.

[44] Laborda F., Jiménez-Lamana J., Bolea E., Castillo J.R. (2013). Critical considerations for the determination of nanoparticle number concentrations, size and number size distributions by single particle ICP-MS. J. Anal. At. Spectrom..

[45] Poitrasson F. (2015). Iron Isotopes. Encyclopedia of Astrobiology.

[46] May T.W., Wiedmeyer R.H. (1998). A Table of Polyatomic Interferences in ICP-MS. At. Spectrosc..

[47] Segura M., Madrid Y., Cámara C. (2003). Elimination of calcium and argon interferences in iron determination by ICP-MS using desferrioxamine chelating agent immobilized in sol–gel and cold plasma conditions. J. Anal. At. Spectrom..

[48] Vidmar J., Oprčkal P., Milačič R., Mladenovič A., Ščančar (2018). Investigation of the behaviour of zero-valent iron nanoparticles and their interactions with Cd^2+^ in wastewater by single particle ICP-MS. Sci. Total Environ..

[49] Russell B., Goddard S.L., Mohamud H., Pearson O., Zhang Y., Thompkins H., Brown R.J.C. (2021). Applications of hydrogen as a collision and reaction cell gas for enhanced measurement capability applied to low level stable and radioactive isotope detection using ICP-MS/MS. J. Anal. At. Spectrom..

[50] Sugiyama N., Nakano K. (2014). Reaction data for 70 elements using O_2_, NH_3_ and H_2_ gases with the Agilent 8800 Triple Quadrupole ICP-MS. Agil. Technol. Tech. Note.

[51] Thor K. (2019). Calcium—Nutrient and messenger. Front. Plant Sci..

[52] Mukherjee B., Weaver J.W. (2010). Aggregation and Charge Behavior of Metallic and Nonmetallic Nanoparticles in the Presence of Competing Similarly-Charged Inorganic Ions. Environ. Sci. Technol..

[53] Wojcieszek J., Jiménez-Lamana J., Ruzik L., Szpunar J., Jarosz M. (2020). To-Do and Not-To-Do in Model Studies of the Uptake, Fate and Metabolism of Metal-Containing Nanoparticles in Plants. Nanomaterials.

[54] Laborda F., Bolea E., Jiménez-Lamana J. (2014). Single particle inductively coupled plasma mass spectrometry: A powerful tool for nanoanalysis. Anal. Chem..

[55] Oprčkal P., Mladenovič A., Vidmar J., Mauko Pranjić A., Milačič R., Ščančar J. (2017). Critical evaluation of the use of different nanoscale zero-valent iron particles for the treatment of effluent water from a small biological wastewater treatment plant. Chem. Eng. J..

[56] Zabetakis K.M., Niño de Guzmán G.T., Torrents A., Yarwood S. (2015). Toxicity of zero-valent iron nanoparticles to a trichloroethylene-degrading groundwater microbial community. J. Environ. Sci. Health A.

[57] Mirlohi S. (2020). In Vitro Evaluation of Iron-Induced Salivary Lipid Oxidation Associated with Exposure to Iron Nanoparticles: Application Possibilities and Limitations for Food and Exposure Sciences. Int. J. Environ. Res. Public Health.

[58] Fedotov P.S., Vanifatova N.G., Shkinev V.M., Spivakov B.Y. (2011). Fractionation and characterization of nano- and microparticles in liquid media. Anal. Bioanal. Chem..

[59] Jreije I., Hadioui M., Wilkinson K.J. (2022). Sample preparation for the analysis of nanoparticles in natural waters by single particle ICP-MS. Talanta.

[60] Wojcieszek J., Jiménez-Lamana J., Bierła K., Ruzik L., Asztemborska M., Jarosz M., Szpunar J. (2019). Uptake, translocation, size characterization and localization of cerium oxide nanoparticles in radish (*Raphanus sativus* L.). Sci. Total Environ..

[61] Chen G., Taherymoosavi S., Cheong S., Yin Y., Akter R., Marjo C.E., Rich A.M., Mitchell D.R.G., Fan X., Chew J. (2021). Advanced characterization of biomineralization at plaque layer and inside rice roots amended with iron- and silica-enhanced biochar. Sci. Rep..

[62] Ehrlich H., Bailey E., Wysokowski M., Jesionowski T. (2021). Forced Biomineralization: A Review. Biomimetics.

[63] Fuente V., Rufo L., Juárez B.H., Menéndez N., García-Hernández M., Salas-Colera E., Espinosa A. (2016). Formation of biomineral iron oxides compounds in a Fe hyperaccumulator plant: *Imperata cylindrica* (L.) P. Beauv. J. Struct. Biol..

