# Supplementary material for: Study of the Stability, Uptake and Transformations of Zero Valent Iron Nanoparticles in a Model Plant by Means of an Optimised Single Particle ICP-MS/MS Method
Source: Nanomaterials (Basel). 2023 May 25;13(11):1736. doi: 10.3390/nano13111736 (PMC10254645; doi:10.3390/nano13111736)
Supplement: Supplementary file 1 [file nanomaterials-13-01736-s001.zip › nanomaterials-2396119-supplementary.pdf]

Table S1. Default instrumental and data acquisition parameters for SP-ICP-MS/MS.

Instrumental parameters	
RF Power	1550 W
Argon gas flow rate	
Plasma	15 L min^{-1}
Auxiliary	0.9 L min^{-1}
Nebulizer	1.10 L min^{-1}
Reaction gas (H_2) flow rate	6 mL min^{-1}
Nebulizer	MicroMist
Skimmer and sampler cones	Pt
Sample depth	8 mm
Sample uptake rate	0.33-0.37 mL min^{-1}
**Data acquisition parameters**	
Data acquisition mode	Time resolved analysis
Dwell time	100 µs
Readings per replicate	1200000
Total acquisition time	120 s
Analyte	Fe
Mass (amu)	56

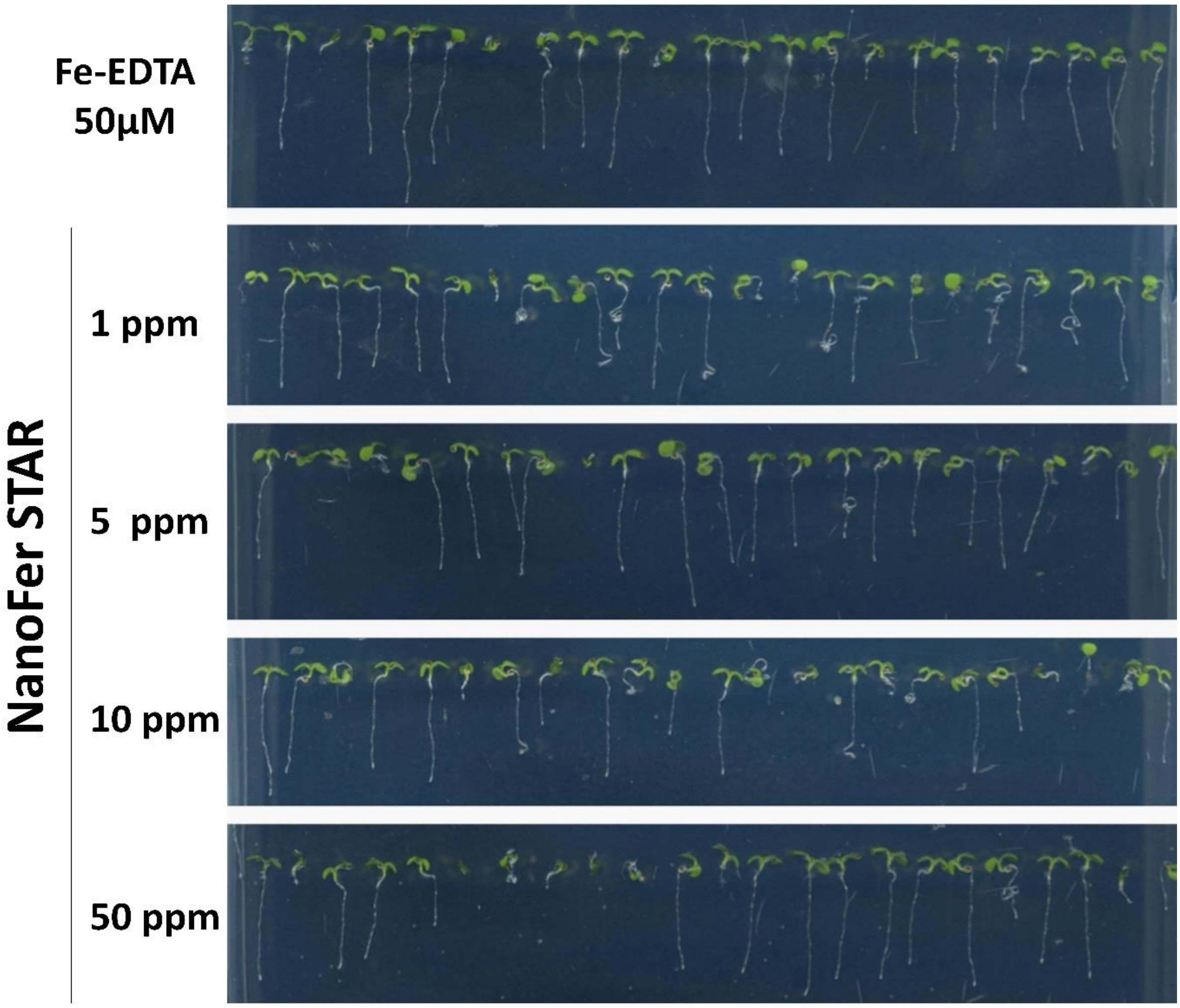

Figure S1. Impact of Nanofer STAR exposure on seedlings grown for 7 days on low concentrations of nZVI.

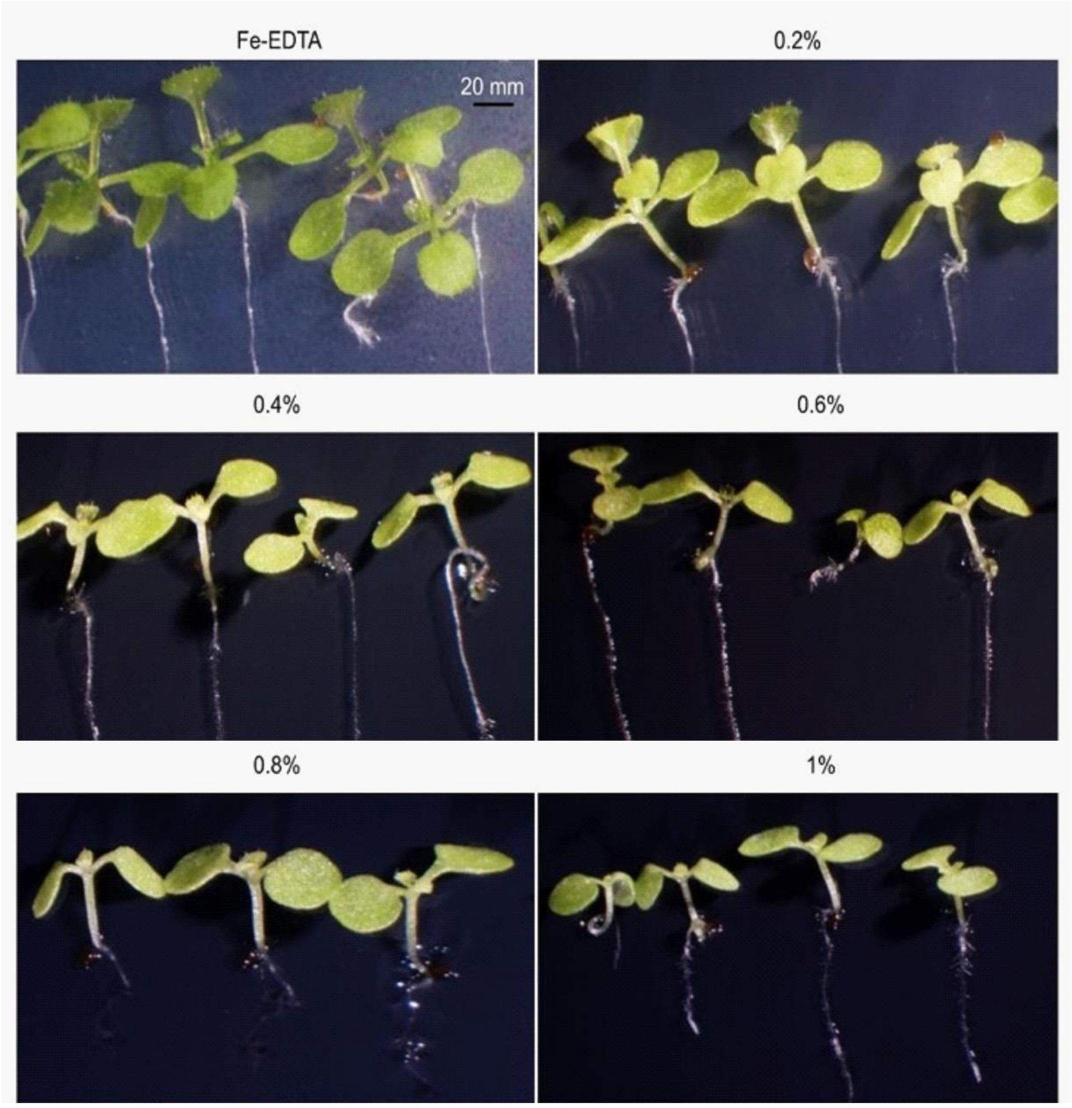

Figure S2. Impact of Nanofer STAR exposure on seedlings grown for 14 days on the indicated nZVI concentrations.

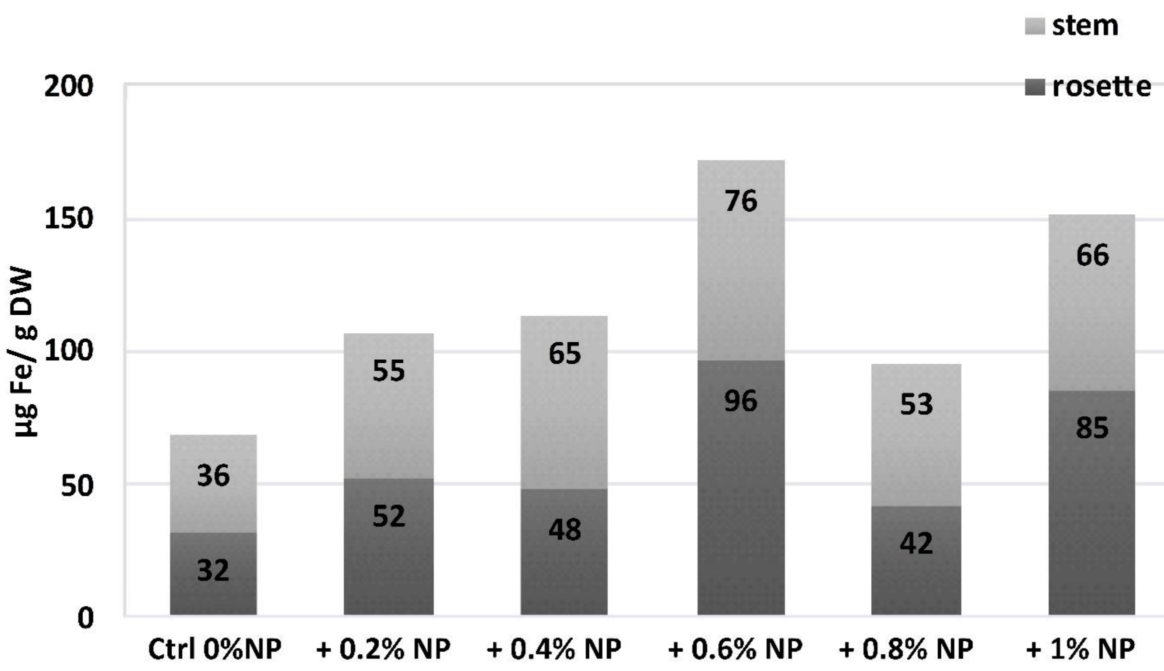

Figure S3. Fe concentration in rosette leaves and floral stem from plants grown on soil supplemented with the indicated concentrations of Nanofer STAR (% nZVI, w/v).

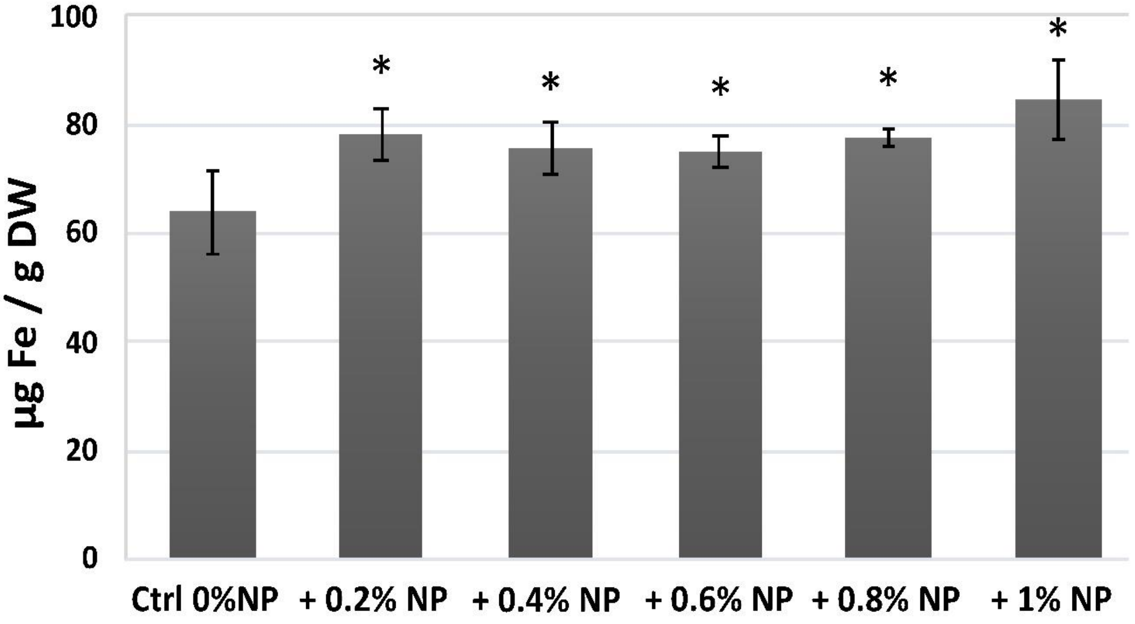

Figure S4. Fe concentration in dry seeds harvested from plants grown on soil supplemented with the indicated concentrations of Nanofer STAR (% nZVI, w/v). Error bars correspond to standard deviation of three replicates, stars indicate significant differences compared to control (Student's test, p<0.1).

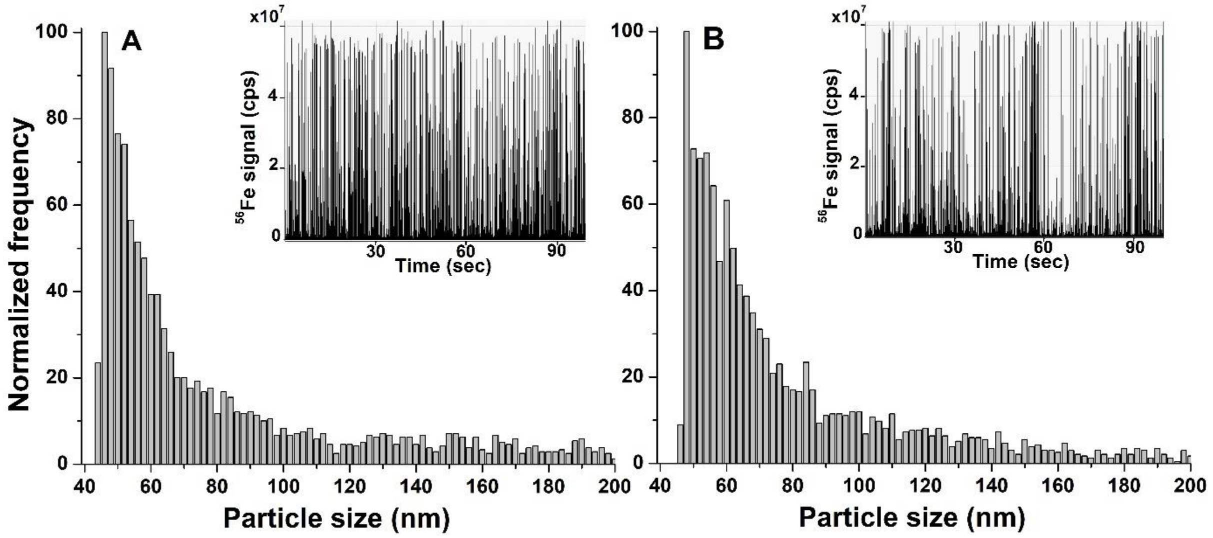

Figure S5. Time scans and size distributions of **(a)** Nanofer 25S and **(b)** Nanofer STAR, obtained after SP-ICP-MS/MS analysis.

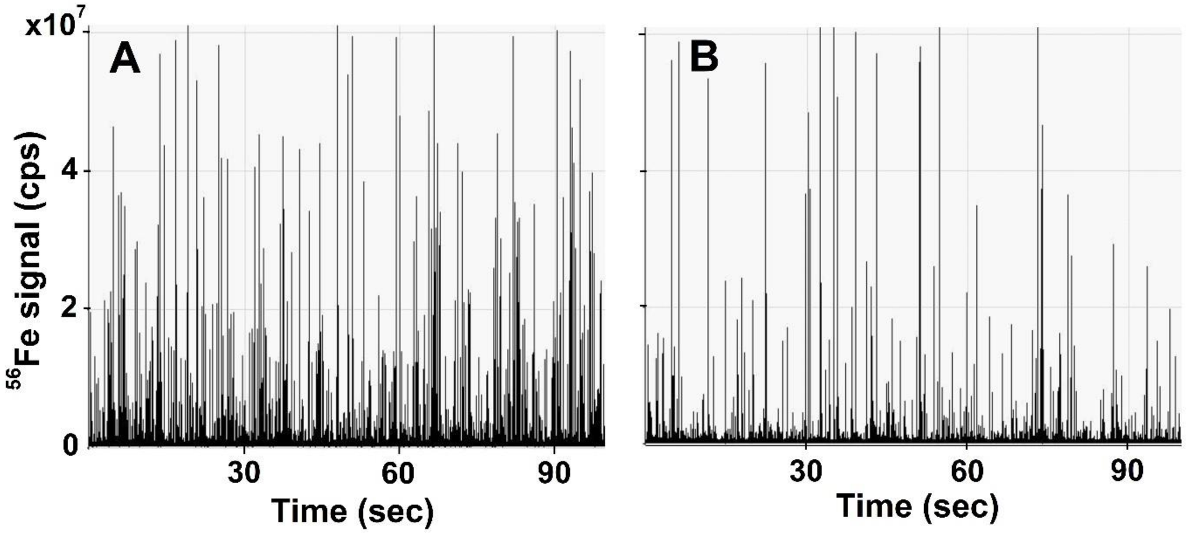

Figure S6. Time scans obtained after SP-ICP-MS/MS analysis of plants treated with **(a)** Nanofer 25S, **(b)** Nanofer STAR.
